# *Fasciolagigantica* Recombinant Bone Morphogenetic Protein (*Fg*BMP) Modulates Various Functions of Buffalo Peripheral Blood Mononuclear Cells

**DOI:** 10.3390/ijms27198552

**Published:** 2026-09-24

**Authors:** Xinping Kong, Sijia Wang, Xiang Gao, Qingyuan He, Weiyu Zhang, Wenda Di

**Affiliations:** 1 College of Animal Science and Technology, Guangxi University, Nanning 530004, China; kxp3802@163.com (X.K.); sijiawang28@163.com (S.W.); 15903868723@163.com (X.G.); xiaohe10595@163.com (Q.H.); 2 National Key Laboratory of Agricultural Microbiology, College of Veterinary Medicine, Huazhong Agricultural University, Wuhan 430070, China

**Keywords:** BMP, *Fasciola gigantica*, immunomodulation, PBMCs

## Abstract

Fasciolosis, a disease induced by liver fluke infection, poses a major threat to the livestock industry. The emergence of drug resistance and the lack of commercial vaccines necessitate the development of novel control strategies. Studies on helminth immunoregulatory molecules have become a key research focus. Bone morphogenetic proteins (BMPs), ligand members of the TGF-β superfamily, have been identified in *Fasciola gigantica* and designated *Fg*BMPs, and are hypothesized to regulate host immune responses. To explore the effects of *Fg*BMP on hosts, the mechanistic actions of *Fg*BMP-1 on buffalo peripheral blood mononuclear cells (PBMCs) were investigated to preliminarily elucidate host–parasite interactions. Molecular docking predicted strong binding between *Fg*BMP-1 and buffalo BMP receptors. Immunostaining using specific monoclonal antibodies revealed the tissue distribution of *Fg*BMP-1, confirming its expression in adult worms, juvenile worms, and excretory–secretory products of *F. gigantica* (*Fg*ESP). Recombinant *Fg*BMP-1 (r*Fg*BMP-1) bound to buffalo PBMCs and significantly suppressed cell proliferation, migration, and phagocytosis while enhancing nitric oxide production. Transcriptional analysis showed that r*Fg*BMP-1 downregulated IFN-γ and IL-10 transcription, whereas it upregulated IL-4 and TGF-β transcription. Transcriptome analysis revealed that r*Fg*BMP-1 may activate the classical TGF-β and MAPK pathways independently or synergistically. Additionally, r*Fg*BMP-1 may modulate host immune responses via crosstalk with the Th17 differentiation and Toll-like receptor signaling pathways. Therefore, *Fg*BMP-1 may mediate immunosuppression in buffalo PBMCs via activation of the TGF-β pathway, thereby refining the theoretical understanding of parasite immune evasion.

## 1. Introduction

Fasciolosis, a disease caused by *Fasciola* species, inflicts substantial economic losses on the livestock industry [1,2]. Currently, triclabendazole (TCBZ) is the drug for the treatment of fasciolosis. However, the emergence of resistance to triclabendazole has necessitated the development of commercial vaccines against *Fasciola gigantica* and *Fasciola hepatica* infections [3,4,5,6]. Elucidating the immunological interactions between *Fasciola* and its host is crucial for the identification of potential vaccine candidates. Helminths are known to modulate the host immune system, leading to pathology through the release of proteins and small molecules that interact with host cells. These helminth-derived molecules play a role in determining the severity of host histopathology and the adaptability of the parasite, mediating parasite survival [7].

The TGF-β signaling pathway is believed to play a role in helminth-mediated immunosuppression of the host [8,9,10]. The high conservation of TGF-β superfamily ligands across species suggests their potential to bind receptors from different hosts [11]. *Fh*TLM can bind to the host-derived TGF-βRII, thereby activating the classical TGF-β pathway, resulting in a decrease in macrophage nitric oxide production and aiding newly excysted juveniles (NEJs) in evading antibody-dependent cell-mediated cytotoxicity (ADCC) [12]. The activin A homologue from *E. multilocularis* (*Em*ACT) can expand host regulatory T cells by augmenting host TGF-β [13]. These findings underscore the significance of helminth-derived TGF-β subfamily ligands in modulating the host immune response. While mammalian-derived BMP ligands exhibit diverse immunomodulatory functions, including Treg shaping, macrophage polarization, monocyte migration and adhesion, and T-cell proliferation [14,15,16,17], the function of helminth-derived BMP ligands in host immune regulation remains unknown.

In the present study, the functions of *Fg*BMP-1 were preliminarily investigated, the presence of *Fg*BMP-1 in the secretory–excretory products of *Fasciola gigantica* (*Fg*ESP) was confirmed, and the immunomodulatory effects of *Fg*BMP-1 on buffalo peripheral blood mononuclear cells (PBMCs) were also explored in vitro. Signal transduction pathways were initially predicted through transcriptomic analysis.

## 2. Results

### 2.1. Amplification of Fgbmp-1 and Bioinformatic Analyses of FgBMP-1

Sequence identity analysis of the C-terminal TGF-β-like domain in BMP ligands revealed distinct conservation patterns between *Fg*BMP-1 and its homologues. *Fg*BMP-1 showed the highest sequence identity with trematode orthologues (*Fh*BMP; 99.02%; *Sm*BMP 91.18%), followed by cestodes (*Em*BMP2: 57.69%) and mammalian hosts (*B*bBMP2 and *Hs*BMP2: both 51.96%; *Mm*BMP4: 50.00%) (Figure 1a). Furthermore, a phylogenetic tree was constructed to elucidate the evolutionary relationships of *Fg*BMP-1 with ligands across diverse species (Figure 1b). As shown in the phylogenetic tree, the BMP and TGF-β subfamilies segregated into two distinct clades.

### 2.2. Homology Modeling of FgBMP-1 and Molecular Docking of FgBMP-1 Binding to Buffalo-Derived Receptors

The 3D structures of the *Fg*BMP-1 precursor and its mature monomer were predicted by homology modeling. The precursor model was constructed based on *F. hepatica* decapentaplegic (A0A4E0RCC7.1.A, 94.23% identity), while the mature monomer was modeled using a TGF-beta family profile domain-containing protein (A0A3S5AZI9.1.A, 80.39% identity). The *Fg*BMP-1 precursor exhibits a more complex and larger ring-shaped structure, in which the anterior region containing LAP envelops the TGF-β like domain (Figure 1c), while the mature monomer of *Fg*BMP-1 exhibits a characteristic hand-like architecture comprising the knuckle, palms, and wrist regions (Figure 1d). (. Structural alignment revealed that both *Bb*BMP-2 (Figure 1e) and *Fg*BMP-1 (Figure 1f) adopt a typical hand-shaped fold (Figure 1g). Furthermore, *Bb*BMP-2 and *Fg*BMP-1 demonstrated high structural similarity, with a root mean square deviation (RMSD) of 0.781.

Molecular docking analysis revealed that *Fg*BMP-1 exhibited strong energy with the majority of host receptors, with values significantly lower than the commonly accepted threshold of −7 kcal/mol (Table 1). *Fg*BMP-1 was predicted to bind stably (≤−7 kcal/mol) to *Bb*BMPR1A (−13.3 kcal/mol), *Bb*BMPR1B (−12.6 kcal/mol), *Bb*BMPR2 (−14.6 kcal/mol), *Bb*ACVR2A (−12.6 kcal/mol), and *Bb*TGFβR2 (−17.9 kcal/mol). In contrast, the binding energy of *Fg*BMP-1 to *Bb*ACVR1 was −5.8 kcal/mol, falling within the intermediate range of −4 to −7 kcal/mol, whereas the binding energy to *Bb*TGFβR1 (−1.5 kcal/mol) was weak, yielding a value higher than the −4 kcal/mol threshold.

### 2.3. rFgBMP-1 Expression and Identification of the Presence of FgBMP-1 in the FgESP

The 720 bp *FgBMP-1* target fragment, encoding the C-terminal TGF-β like domain, was amplified and subsequently ligated into a 5074 bp linearized pET-28a(+) vector. The recombinant construct was then verified by sequencing. Subsequently, the recombinant vector was transferred into the *E. coli* BL21 (DE3), and protein expression was induced with 0.1 mM IPTG at 37 °C for 12 h to produce r*Fg*BMP-1. SDS-PAGE analysis revealed a specific band at approximately 35 kDa in the cell lysate with induction, consistent with the predicted molecular mass of 32.18 kDa (Figure 2a). Following denaturation, purification, and renaturation procedures, the target protein r*Fg*BMP-1 was successfully obtained (Figure 2b).

As shown in Figure 2c, the mouse anti-*Fg*BMP-1 monoclonal antibody specifically recognized a band at ~25 kDa in the *Fg*ESP sample.

### 2.4. Immunohistological Analysis of FgBMP-1 Distribution in F. gigantica

The mouse anti-*Fg*BMP-1 antibody specifically recognized native *Fg*BMP-1 in *F. gigantica* adult extract and in vitro-expressed r*Fg*BMP-1. The pre-immune serum showed no reactivity to either. Subsequently, the antibody and pre-immune serum were used to localize the native *Fg*BMP-1 in adults and 6-week juveniles of *F. gigantica*. In adults, *Fg*BMP-1 was localized to the vitellaria, testis, cecum, and tegument (Figure 2d). In 6-week juveniles, *Fg*BMP-1 was detected in the parenchyma, cecal contents, and tegument. No positive signals were observed in worms treated with pre-immune serum (Figure 2e).

### 2.5. rFgBMP-1 Binds to PBMCs and Suppresses Their Proliferation, Migration, and Phagocytosis while Enhancing NO Production

The binding of r*Fg*BMP-1 to PBMCs was assessed by indirect immunofluorescence. As shown in Figure 3a, distinct fluorescent signals were observed within the cell membrane and cytoplasm of PBMCs stimulated with r*Fg*BMP-1 (10 µg/mL) and further incubated with anti-r*Fg*BMP-1 primary antibody followed by Cy3-conjugated secondary antibody (Figure 3a, bottom panel). The control group consisted of PBMCs incubated with anti-r*Fg*BMP-1 primary antibody and secondary antibody (no r*Fg*BMP-1 stimulation), which showed no detectable fluorescence (Figure 3a, top panel).

The effects of r*Fg*BMP-1 on the proliferation of PBMCs were evaluated using a CCK-8 assay. As shown in Figure 3b, compared to the untreated group, ConA (10 μg/mL) promoted PBMC proliferation (*p* < 0.0001), whereas r*Fg*BMP-1 suppressed it at 20 μg/mL (*p* < 0.01), 40 μg/mL (*p* < 0.001), and 80 μg/mL (*p* < 0.001).

As shown in Figure 3c, r*Fg*BMP-1 significantly inhibited PBMC migration at 5 (*p* < 0.05), 10, 20, 40, and 80 μg/mL (*p* < 0.0001) compared to the negative control.

Phagocytic activity was determined by measuring the percentage of FITC-labeled dextran in PBMCs by flow cytometry. Compared with the control group, r*Fg*BMP-1 reduced the phagocytosis rates of PBMCs at 10 (*p* < 0.05), 40 (*p* < 0.0001), and 80 μg/mL (*p* < 0.0001) (Figure 3d).

As shown in Figure 3e, r*Fg*BMP-1 significantly enhanced NO production at 5, 10, 20, 40, and 80 μg/mL (*p* < 0.0001).

### 2.6. rFgBMP-1 Inhibited the Apoptosis and Affected Cytokine Transcription

As shown in Figure 3f, r*Fg*BMP-1 significantly enhanced transcription of the anti-apoptotic gene *Bcl-2* at 10, 20, 40, and 80 μg/mL (*p* <0.01) (Figure 3f). Conversely, r*Fg*BMP-1 significantly downregulated transcription of the pro-apoptotic gene *Bax* at 5, 10, 20, 40, and 80 μg/mL (*p* < 0.001) (Figure 3g).

The effects of r*Fg*BMP-1 on the transcription of cytokines in PBMCs were evaluated by qPCR (Figure 4). r*Fg*BMP-1 significantly upregulated transcription of IL-4 and TGF-β at 20, 40, and 80 μg/mL (Figure 4a and Figure 4d). Furthermore, r*Fg*BMP-1 downregulated IFN-γ transcription at all tested concentrations and inhibited IL-10 transcription at 40 and 80 μg/mL (Figure 4b,c).

### 2.7. RNA-Seq Analysis of rFgBMP-1–Stimulated PBMCs Reveals Transcriptomic Profiling and Differentially Expressed Genes 

To identify the potential signaling pathways involved, the cellular transcriptome was analyzed. As depicted in Figure 5a, the control group and r*Fg*BMP-1-treated group shared 6134 genes, with the r*Fg*BMP-1-treated group having 1061 unique genes and the control group having 4220 unique genes. Comparative analysis identified 7145 differentially expressed genes (DEGs), comprising 2171 significantly upregulated and 4974 significantly downregulated (Figure 5b).

GO enrichment analysis was performed to predict the biological functions of the differentially expressed genes, and the top 10 terms with the most significant enrichment in the biological process (BP), cellular component (CC), and molecular function (MF) categories are displayed based on adjusted *p*-values (Figure 5c).

KEGG pathway enrichment analysis was performed, and the 20 most significant terms were listed and ranked based on padj values (Figure 5d). Among those, the MAPK pathway showed the highest gene ratio. In addition, the classical and non-classical TGF-β pathway, together with the MAPK and PI3K-Akt signaling pathways, were identified as enriched. Notably, within the classical TGF-β pathway, ligands (BMP-5, BMP-7 and TGF-β1), receptors (BMPR2, ACVR1B, TGFβR1, TGFβR2, AMHR2), SMAD proteins (Smad5, Smad2, Smad7, Smad3), and regulatory genes and transcription factors were also identified in the classical TGF-β pathway (Figure 5e).

A protein–protein interaction network was constructed to uncover the signaling pathways associated with the interacting proteins encoded by differentially transcribed genes identified in the classical TGF-β pathway. As depicted in Figure 5f, the components of the classical TGF-β pathway, which include BMP-7, BMPR2, TGFβ1, TGFβR1, TGFβR2, Smad3, and Smad7, can regulate pathways individually or synergistically. TGFβ1, TGFβR1, TGFβR2, and SMAD3 were associated with multiple pathways, including the AGE-RAGE signaling pathway in diabetic complications, cellular senescence, Th17 cell differentiation, and the FoxO signaling pathway. Furthermore, osteoclast differentiation and the MAPK signaling pathway were also found to be regulated by TGFβ1, TGFβR1, and TGFβR2.

## 3. Discussion

In the present study, *Fg*BMP-1 was functionally characterized. Through interaction with host cells, this protein activates TGF-β and MAPK signaling pathways, thereby modulating the proliferation, migration, and apoptosis of host PBMCs. Collectively, these immunomodulatory effects likely facilitate the establishment of a favorable microenvironment for the survival and development of *Fasciola gigantica*. *Fg*BMP-1 contains seven conserved cysteine residues, a characteristic feature of BMP ligands that distinguishes them from TGF-β ligands, which typically contain nine such residues. Sequence alignment revealed that *Fg*BMP-1 shares 50–52% identity with mammalian homologues, 56.73–58.65% with cestode homologues, and 83.33–99.02% with other trematode homologues. Phylogenetic analysis showed that the BMP branch was clearly distinct from the TGF-β branch and could be further subdivided into clades corresponding to the phyla Platyhelminthes, Nematoda, and Chordata. Furthermore, 3D modeling revealed that the mature *Fg*BMP-1 monomer shares high structural similarity with its buffalo homologue (*Bb*BMP-2), exhibiting significant overlap in spatial conformation, highlighting the evolutionary conservation of BMP ligands across species. Based on the conservation of secondary and tertiary structures, we hypothesize that *Fg*BMP-1 may bind to buffalo receptors; however, this requires further experimental verification.

Given that Wu reported higher transcription abundance of *Fg*BMP-1 in cercariae, metacercariae, 6 week-old juveniles, 10 week-old juveniles, and adults [18,19], the tissue localization of native *Fg*BMP-1 in 6-week-old juveniles and adults was investigated. The presence of *Fg*BMP-1 in the cecum, vitellaria, tegument, and testis suggests that it may be involved in nutrient digestion and absorption, reproductive organ development, and egg-laying processes. In 6-week-old juveniles, *Fg*BMP-1 is predominantly localized in the parenchymal tissues, whereas in adult worms it is concentrated in the reproductive system. This distribution suggests that during the juvenile stage, the protein primarily serves to maintain essential physiological functions and modulate host immune responses. Upon sexual maturity, the protein’s localization shifts to the reproductive organs, implying a functional transition from supporting somatic growth to regulating reproductive processes.

Helminths secrete and excrete various components during the parasitic stage, which consist of soluble ESPs and insoluble extracellular vesicles (EVs). Among these, EVs serve as critical carriers of non-classically secreted proteins, transporting proteins derived from the tegument or gastrodermal cells to the host [20]. Cwiklinski et al. suggested that the larger 15 K EVs of *F. hepatica* are released from the gastrodermal cells, whereas the smaller 120K EVs population originates from multivesicular bodies within the tegumental syncytium [21,22]. Given that *Fg*BMP-1 lacks a signal peptide and transmembrane domain, it is inferred to be a non-classically secreted protein that may utilize EVs to interact with host cells [20,21]. *F. hepatica* EVs are released from the tegumental syncytium via multivesicular bodies, as demonstrated by Cwiklinski et al. [21]. By analogy, *Fg*BMP-1 localization in the tegument and cecum—tissues capable of synthesizing EVs—supports the hypothesis that *Fg*BMP-1 may be sorted into multivesicular bodies and potentially released via small exosome-like vesicles; however, this remains to be experimentally validated.

The persistence of chronic parasitic infections often depends on the parasites’ ability to effectively interfere with the proliferation and migration of the host’s lymphocytes. Typically, antigen recognition by lymphocytes triggers rapid clonal expansion and effector cell migration [23]; however, *Fasciola* species employ immune evasion strategies. For instance, components like *Fh*Teg and *Fh*ESP induce T-cell anergy via dendritic cells or macrophages, thereby suppressing downstream cellular responses and facilitating parasite persistence [24,25,26]. Here, we demonstrate that *Fg*BMP-1 significantly inhibits the proliferation and migration of lymphocytes within buffalo PBMCs in vitro. This impairment likely restricts immune cell recruitment to parasitic niches, thereby diminishing tissue repair efficacy and anti-parasitic killing capacity. Notably, in contrast to its inhibitory effect on lymphocytes, r*Fg*BMP-1 promoted the proliferation and differentiation of monocytes into macrophages (unpublished data). Given the established immunosuppressive capacity of macrophages on lymphocytes [27], we hypothesize that r*Fg*BMP-1 mediates the observed lymphocyte suppression by driving macrophage expansion. Further studies are warranted to determine the specific lymphocyte subsets targeted by this regulatory mechanism and the underlying signaling pathways.

While apoptosis is fundamental to host homeostasis, helminths frequently co-opt this process to establish immunosuppression [28]. *F. hepatica* infection and its excretory–secretory products (ESPs) are well documented to induce leukocyte apoptosis, targeting eosinophils, macrophages, and PBMCs [29,30]. Similarly, Schistosoma infections and soluble egg antigen (SEA) drive CD4+ T-cell apoptosis via the Fas pathway [31,32]. Contrary to this prevailing paradigm where helminth-derived products predominantly trigger host cell apoptosis, our data reveal a divergent regulatory mechanism. In vitro stimulation of buffalo PBMCs with r*Fg*BMP-1 upregulated the anti-apoptotic gene *Bcl-2* while downregulating the pro-apoptotic gene *Bax*. We hypothesize that this inhibition of immune cell apoptosis suppresses Th1-mediated inflammation and cytokine release, thereby attenuating anti-parasitic immunity and facilitating the establishment of *F. gigantica*.

Nitric oxide (NO) serves as a pivotal mediator of antibody-dependent cell-mediated cytotoxicity (ADCC), a process wherein host macrophages, primed by antibodies, generate cytotoxic levels of NO via inducible nitric oxide synthase (iNOS) to eliminate helminths [33,34,35,36]. Beyond its direct antiparasitic effects—compromising tegument integrity, somatic tissues, and mitochondrial function—NO also limits excessive immunopathology by modulating granuloma formation [36]. While *F. gigantica* ESP components (e.g., 14-3-3ε, Rab-10) are known to induce NO production, NO exhibits a functional duality: at high concentrations, NO promotes apoptosis to facilitate the clearance of effete cells and resolve inflammation [37], yet it concurrently exerts broad immunosuppressive effects, including the inhibition of Th1/Th2 responses [35,38,39]. In this study, r*Fg*BMP-1 significantly upregulated NO production in buffalo PBMCs in vitro. These data suggest that r*Fg*BMP-1 may orchestrate a multifaceted immunomodulatory network, potentially linking ADCC-mediated cytotoxicity with the suppression of lymphocyte proliferation, modulation of cytokine profiles, and the induction of apoptosis. However, given the concurrent cytokine shifts—elevated IL-4 and TGF-β alongside reduced IFN-γ—the r*Fg*BMP-1-induced NO in PBMCs likely reflects a mixed M1–M2-polarizing signal wherein the net immunological outcome is immune dampening rather than parasite clearance.

The efficient clearance of apoptotic cells—efferocytosis—is essential for maintaining tissue homeostasis, as it prevents secondary necrosis and the subsequent release of immunogenic intracellular contents [40,41,42]. This process relies on specialized phagocytes, such as macrophages, which sequentially interpret “find-me” cues to localize targets and recognize “eat-me” cues to mediate precise engulfment [43]. In our transwell assays, r*Fg*BMP-1 significantly impaired the phagocytic activity of buffalo PBMCs, as measured by FITC-dextran uptake. We postulate that this defect stems from a dual mechanism: first, the r*Fg*BMP-1-induced inhibition of apoptosis likely diminishes phagocytic signals from target cells; and second, the concomitant suppression of PBMC migration prevents effector cells from accessing potential clearance sites.

Helminth infections orchestrate complex immunomodulatory networks within the host. While an initial Th1/M1-biased inflammatory response (characterized by IFN-γ and TNF-α) is mounted against invading parasites, successful pathogens rapidly subvert this by promoting a Th2–M2 shift to limit immunopathology and ensure persistence [44,45]. Consistent with this paradigm, our data demonstrate that r*Fg*BMP-1 significantly downregulated IFN-γ expression while upregulating IL-4 transcription in buffalo PBMCs. This cytokine polarization suggests that r*Fg*BMP-1 actively suppresses pro-inflammatory responses, thereby attenuating anti-parasitic killing and mitigating tissue damage. Furthermore, the concurrent upregulation of TGF-β implies that r*Fg*BMP-1 may drive the expansion or activation of regulatory T cells (Tregs) and/or M2 macrophages—key arbiters of chronic immune tolerance. Interestingly, we observed a concomitant decrease in IL-10 transcription. Since IL-10 production is not universal among all immunosuppressive subsets, this reduction may reflect the selective induction of a distinct regulatory phenotype by r*Fg*BMP-1—one potentially independent of classical IL-10 signaling. Indeed, certain helminth-derived molecules, such as the *Acanthocheilonema viteae* cysteine protease inhibitor AV17, selectively induce macrophage-mediated suppression without engaging Tregs [46]. Thus, the r*Fg*BMP-1-mediated inhibition of PBMC proliferation and the observed IL-10 dynamics likely signify the activation of a unique immunoregulatory axis tailored to facilitate *F. gigantica* chronicity.

To delineate the immunomodulatory landscape of r*Fg*BMP-1 in water buffalo, we performed transcriptomic profiling on treated PBMCs. Gene Ontology (GO) analysis revealed significant enrichment in biological processes governing apoptosis and cell proliferation, aligning with the established functions of BMP ligands and validating our in vitro phenotypic observations. Kyoto Encyclopedia of Genes and Genomes (KEGG) pathway analysis further highlighted enrichment in oncogenic signaling, including the “Pathways in cancer” (which exhibited the highest gene ratio), PD-L1–PD-1 checkpoint interactions, choline metabolism, and central carbon metabolism, suggesting that *Fg*BMP-1 may profoundly influence host cellular signaling networks. However, it should be explicitly noted that the enrichment of oncogenic pathways in PBMCs is most likely a bystander effect resulting from the co-activation of MAPK and TGF-β signaling cascades, rather than evidence of tumorigenic transformation. Therefore, this observation should not be over-interpreted as an indication of malignant progression. Concurrently, enrichment of immune-centric pathways—such as Th17 differentiation and TNF, BCR, and TLR signaling—indicated that r*Fg*BMP-1 broadly targets diverse immune cell lineages. Notably, the TGF-beta and MAPK signaling pathways were among the most significantly enriched. We propose a model wherein TGF-β signaling interacts with the MAPK axis to establish systemic immune tolerance. This is mechanistically supported by the downregulation of genes involved in T-cell activation (TLRs and co-stimulatory molecules) and the suppression of leukocyte chemotaxis via inhibition of CXCL and CCL family members downstream of the IL-17 and TNF pathways. Analysis of the TGF-β superfamily cascade confirmed that r*Fg*BMP-1 engages both classical branches: the BMP branch (phosphorylating Smad1/5/8) and the TGF-β/activin branch (phosphorylating Smad2/3). Molecular docking simulations revealed high binding affinity between *Fg*BMP-1 and the buffalo type II receptors BMPR2 and TGFβR2, with comparatively lower affinity for the type I receptors ACVR1 and TGFβRI. This suggests that r*Fg*BMP-1 may preferentially engage type II receptors BMPR2/TGFβR2, potentially stabilizing a trimolecular complex necessary for type I receptor activation; however, this prediction requires experimental validation (e.g., SPR or co-IP). However, an indirect antagonistic effect—whereby *Fg*BMP-1 interferes with endogenous ligand–receptor interactions—cannot be excluded. Given the distinct transcriptional kinetics of *F. gigantica* TGF-β superfamily ligands (e.g., BMP, BMP-3, TLM, TLP) across developmental stages [18] (Wu et al., 2023 [19]), the immunomodulatory effect of *Fg*BMP-1 likely varies in space and time in vivo. Future studies are needed to ascertain whether r*Fg*BMP-1 acts as a competitive agonist at the TGF-β receptor complex to drive differential Smad phosphorylation, and how its tissue-specific accumulation correlates with parasite migration and immune evasion.

## 4. Materials and Methods

### 4.1. Parasites and Animals

*Fasciola gigantica* adult worms were collected from the bile ducts of naturally infected water buffaloes (*Bubalus bubalis*) slaughtered at a local abattoir in Nanning City, South China. Buffaloes were selected based on the presence of fluke eggs in fecal samples examined by the sedimentation method. Adult worms were recovered by dissecting the bile ducts, washed extensively in prewarmed (37 °C) PBS (pH 7.4), and identified morphologically and molecularly. Total genomic DNA was extracted from individual worms, and the internal transcribed spacer 2 (ITS-2) region was amplified and sequenced. PCR products were purified, sequenced, and compared with reference sequences in GenBank to confirm the species as *F. gigantica*. Metacercariae were obtained from experimentally infected freshwater snails (*Galba pervia*) and used to prepare 6-week juveniles. Six-week juveniles were recovered from experimentally infected KM mice orally inoculated with viable metacercariae. At 6 weeks post-infection, animals were euthanized, livers were teased in prewarmed PBS, juvenile flukes were harvested from the liver parenchyma, washed, and either used immediately or stored at −80 °C. All parasite stages were confirmed as *F. gigantica* by ITS-2 analysis.

The buffaloes that served as blood donors in this study were from the Buffalo Research Institute, Chinese Academy of Agricultural Sciences, and were verified negative for *Fasciola* infection by serological detection. BALB/c mice (6–8 weeks old) purchased from Guangxi Medical University were used for monoclonal antibody preparation. Animal study, including serum collection, was approved by the Ethics Committee of the School of Animal Science and Technology at Guangxi University. All animal handling in this study adhered to the principles of good animal practices required by the Animal Ethics Procedures and Guidelines of the People’s Republic of China.

### 4.2. Molecular Modeling and Affinity Prediction of FgBMP-1 to Buffalo-Derived Receptors

The total RNA of *F. gigantica* was extracted using a Trizol kit (19201ES60, Yeasen, Shanghai, China), and RNA concentration and purity were measured. Then cDNA was synthesized using HiScript II Q RT SuperMix (R223-01, Vazyme, Nanjing, China) and the synthesized cDNA was stored at −80 °C. Primers were designed to clone the coding sequence (CDS) of *Fg*BMP-1 (see Supplementary Materials (Table S1)), and the resulting PCR products were sequenced (Sangon Biotech Company, Guangzhou, China). Multiple-sequence alignment was conducted using MEGA7 using default parameters. Phylogeny was determined via the maximum likelihood method and validated using 1000 bootstrap replicates in MEGA7.

Homology modeling of *Fg*BMP-1 was carried out utilizing SWISS-MODEL, and credibility was evaluated by Verification Server SAVES. *Bb*BMP-2, the buffalo (*Bubalus bubalis*) BMP homologue most closely related to *Fg*BMP-1, was selected as the template for homology modeling. Then, alignment of the 3D structures between *Fg*BMP-1 and *Bb*BMP-2 was performed, and RMSD (root mean square deviation) was calculated using PyMol 3.1 to compare their structural similarity quantitatively. Homology modeling of buffalo-derived BMP receptors was constructed using the SWISS-MODEL and verified. Then, molecular docking between *Fg*BMP-1 and the receptors was conducted using the GRAMM Docking Web Server, and the docking results were visualized with PyMol software.

### 4.3. FgBMP-1 and Monoclonal Antibody Production

Primers (*Fg*BMP1-2F/2R and pET28a-F/R) were designed to amplify the conserved domain of *Fg*BMP and linearize the vector, respectively. Then, the PCR product was ligated into the pET-28a(+) vector and transferred into *Escherichia coli* (DH5α) following the instructions of the ClonExpress^®^ II One Step Cloning Kit (C112-01, Vazyme, Nanjing, China). Positive clones were sequenced and were transferred into *E. coli* BL21 (DE3), resulting in the construct pET-28a(+)-*Fg*BMP-1. Recombinant *Fg*BMP-1 (r*Fg*BMP-1) was induced with 0.1 mM isopropyl β-D-1-thiogalactopyranoside (IPTG) and incubated for 16 h at 37 °C 150 rpm. Bacteria were harvested subjected to sonication, followed by solubilization of the inclusion bodies in 8 M urea and purification using the His-tagged protein purification kit (CW0893S, CWBIO, Taizhou, China). The refolding of the protein was achieved by gradually dialyzing from an 8 M urea solution to PBS buffer at 4 °C. The purified r*Fg*BMP-1 was treated with a ToxinEraser™ Endotoxin Removal Kit (L00338, GenScript, Nanjing, China) to eliminate the endotoxin, and endotoxin levels were determined using a ToxinSensor™ Chromogenic LAL Endotoxin Assay Kit (L00350C, GenScript, Nanjing, China). After filtration sterilization with a 0.22 μm filter, the concentration of r*Fg*BMP-1 was measured using a BCA protein concentration determination kit (PC0020, Solarbio, Beijing, China), and stored at −80 °C.

Six BALB/c mice (6–8 weeks old) were subcutaneously immunized with 50 μg r*Fg*BMP-1 in complete Freund’s adjuvant, followed by two booster injections with 50 μg of r*Fg*BMP-1 in incomplete Freund’s adjuvant at two-week intervals. Then, serum was collected from the tail veins of the mice 7 days after the final booster, and serum antibody titers were detected using indirect ELISA. Mice with the highest serum titer were selected for intraperitoneal booster immunization and subsequent cell fusion. After 3 days, spleen cells from immunized mice were collected and fused with mouse myeloma Sp2/0 cells. The supernatant of hybridoma cells was collected and screened using indirect ELISA and Western blot to identify positive hybridoma clones capable of recognizing both the r*Fg*BMP-1 and native *Fg*BMP-1 in *F. gigantica* adult. Three positive hybridoma cell lines were expanded and cultured, and the cell supernatants were used to identify the antibody subtypes. Specific hybridomas were collected and injected into Freund adjuvant-primed BALB/c mice to produce ascites fluid. Antibodies were purified from the ascites fluid using protein G, and their antibody titers were tested using indirect ELISA.

### 4.4. Western Blot Detection and Immunolocalization

*Fg*ESP was prepared as previously described [47]. Briefly, 30 μg denatured *Fg*ESP was subjected to electrophoresis and transferred to a PVDF membrane. The membrane was then blocked with TBST containing 5% skim milk for 2 h and incubated with the mouse anti-*Fg*BMP-1 monoclonal antibody overnight at 4 °C. After washing, the membrane was incubated with HRP-conjugated goat anti-mouse IgG (CW0102S, CWbio, Taizhou, China) for 1 h at 37 °C, followed by 3 washes with PBST, then bands were visualized on an ImageQuant LAS 500 (GE Healthcare, Beijing, China).

To ensure the specificity of anti-*Fg*BMP-1 monoclonal antibodies, anti-*Fg*BMP-1 monoclonal antibody and mouse pre-immune serum were used to recognize the native *Fg*BMP-1 in *F. gigantica* adult by Western blot. *F. gigantica* adult fluke extracts were prepared using a Whole Protein Extraction Kit (BC3710, Solarbio, Beijing, China), and the Western blot was performed as described above. Then, immunolocalization was performed in juveniles and adults of *F. gigantica*. The active 6-week juveniles and adults of *F. gigantica* were fixed in 4% paraformaldehyde for 24 h, followed by dehydration in a series of ethyl alcohol, benzene, and xylene solutions with increasing concentrations (75%, 85%, 90%, 95%, 100%). The fluke was then embedded in paraffin at 65 °C. Then, paraffin blocks were sliced and soaked in 0.01 M citrate buffer (pH 6.0), heated to 95 °C for 10 min, and then washed with PBS for 15 min upon cooling. After blocking with 5% BSA for 15 min, the slices were incubated overnight with the anti-*Fg*BMP-1 antibody and mouse pre-immune serum at 4 °C, then incubated for a further 1 h at room temperature with FITC-conjugated goat anti-mouse IgG (Proteintech, SA00003-1, Wuhan, China). After incubation with DAPI for 15 min at room temperature and three washes with PBS, the tissue slices were mounted in anti-fade mounting medium and visualized under a fluorescence microscope (Z2, Zeiss, Oberkochen, Germany).

### 4.5. Immunofluorescence

Peripheral blood mononuclear cells (PBMCs, 1 mL) from buffalo (1 × 10^6^ cells/mL) were seeded into a 12-well plate and stimulated with 10 µg/mL r*Fg*BMP-1 or without r*Fg*BMP-1 (as a control) for 24 h at 37 °C with 5% CO_2_. Following the removal of non-adherent PBMCs, the adherent PBMCs were washed with PBS and fixed with 4% paraformaldehyde for 30 min and washed with PBS three times. For the r*Fg*BMP-1-treated group, PBMCs were incubated with anti-r*Fg*BMP-1 monoclonal antibody overnight at 4 °C, followed by Cy3-labeled goat anti-mouse IgG secondary antibody. For the control, PBMCs were incubated with the primary anti-r*Fg*BMP-1 antibody and Cy3-labeled secondary antibody, omitting r*Fg*BMP-1 stimulation. All other incubation and washing steps were identical between groups. Finally, the PBMC-contained slides were taken out and buckled upside down on another glass slide containing the anti-quenching agent. The binding of r*Fg*BMP-1 to PBMCs was detected by a fluorescence microscope (Z2, Zeiss, Oberkochen, Germany).

### 4.6. Cell Proliferation and Migration Assay

PBMC suspension (100 μL, 1 × 10^6^ cells/mL) was seeded into a 96-well plate and stimulated with various concentrations of r*Fg*BMP-1 (5, 10, 20, 40, and 80 µg/mL). Concanavalin A (ConA) at 10 µg/mL served as a positive control, while PBS was used as a negative control. The plate was incubated at 37 °C for 48 h. Then, 10 µL of CCK-8 solution was added to each well and incubated for 4 h at 37 °C in the dark, and absorbance at 450 nm was measured with a microplate reader. Cell viability (%) = (cell viability of treated group/cell viability of negative control group) × 100%. The assay was carried out 3 times.

Two milliliters of PBMC suspension (1 × 10^6^ cells/mL) was seeded into a 6-well plate and stimulated with different concentrations of r*Fg*BMP-1 (5, 10, 20, 40 and 80 µg/mL) or without r*Fg*BMP-1 protein (as a control) at 37 °C for 48 h. The PBMCs were collected by digestion with 0.25% trypsin (Gibco, Grand Island, NY, USA) and centrifugation, and subsequently adjusted to a concentration of 1 × 10^6^ cells/mL with complete RPMI-1640 medium. One hundred microliters of PBMC suspension and 600 μL complete RPMI-1640 medium were added to the upper chamber and lower chamber of transwell (3422, Corning, Corning, NY, USA), respectively, and incubated at 37 °C with 5% CO_2_ for 4 h. Finally, the PBMCs that migrated to the lower chamber were counted. The cell migration rate was calculated using the formula Cell migration rate  = (total PBMCs recovered from the lower chamber/total number of PBMCs added to the upper chamber) × 100%. The assay was carried out 3 times.

### 4.7. Phagocytosis and Nitric Oxide Production Assay

PBMC suspension (2 mL, 1 × 10^6^ cells/mL) was seeded into a 6-well plate and stimulated with different concentrations of r*Fg*BMP-1 (5, 10, 20, 40 and 80 µg/mL) or left untreated as a control for 24 h at 37 °C. Subsequently, the supernatant was discarded, and the adherent cells were washed twice with PBS. Then, 1 mL of FITC–dextran was added to each well and incubated for 1 h at 37 °C in the dark. Then, the FITC–dextran solution was removed. The cells were collected by trypsin digestion and centrifugation at 2000 rpm for 5 min. Then, the cells were resuspended in 200 µL of PBS. Finally, flow cytometry was performed to detect the phagocytic ability of PBMCs using an Attune^®^ NxT Acoustic Focusing Flow Cytometer (Life Technologies, Carlsbad, CA, USA). FlowJo-v10.8.1 software was used to analyze flow cytometry data and calculate phagocytosis percentage. The assay was carried out 3 times.

Then, 1 mL of PBMC suspension (1 × 10^6^ cells/mL) was seeded into a 12-well plate and stimulated with different concentrations of r*Fg*BMP-1 (5, 10, 20, 40 and 80 µg/mL) or without r*Fg*BMP-1 (as a control) at 37 °C at 5% CO_2_ for 24 h. Cell supernatants were collected and centrifuged to discard the cell pellet. The concentration of NO was detected using a nitric oxide detection kit (Beyotime, S0021S, Shanghai, China) according to the manufacturer’s instructions. The absorbance at 540 nm was measured with a microplate reader, and nitric oxide content was calculated based on the established standard curve. The assay was carried out 3 times.

### 4.8. Real-Time Quantitative PCR (qPCR) Test

PBMC suspension was stimulated with different concentrations of r*Fg*BMP-1 (5, 10, 20, 40 and 80 µg/mL) or without r*Fg*BMP-1 (as a control) at 37 °C for 24 h. Then, cells were collected and the supernatant was discarded by centrifugation. RNA extraction and cDNA synthesis were performed as previously described [48]. The transcript abundance of cytokines (IFN-γ*,* IL-4, IL-10, TGF-β) and apoptosis-related genes (*Bcl-2, Bax*) was determined by quantitative PCR using ChamQ Universal SYBR qPCR Master Mix (Q711-02, Vazyme, Nanjing, China). GAPDH was used as a reference, and the relative transcription value was calculated by the 2 ^(−ΔΔCt) method. All primers are listed in the Supplementary Materials (Table S1). The assay was carried out 3 times.

### 4.9. Transcriptomic Analysis

PBMCs stimulated with 40 µg/mL r*Fg*BMP-1 for 24 h served as the experimental group, and unstimulated PBMCs served as the control group. RNA was extracted from both groups, and the integrity and total amount of the RNA were detected using an Agilent 2100 bioanalyzer. Then, RNA libraries were constructed and high-throughput sequencing was performed. After filtering out reads with adapters, unrecognized reads, and low-quality reads, clean data were obtained. Afterwards, quality assessments were conducted on the clean data, including Q20, Q30, and GC content calculations. Next, the clean reads were aligned to the *Bubalus bubalis* reference genome using HISAT2 2.1 for gene annotation. Gene transcription levels were evaluated using the feature counts tool in Subread 2.1, with FPKM as the expression metric. In order to screen the differentially expressed genes, the raw read counts of the samples were standardized to eliminate the influence of technical differences and other irrelevant factors. The *p*-value for each gene was calculated, and the FDR value was obtained through multiple hypothesis testing correction. Differentially expressed genes were screened based on the criteria (log2(FoldChange) ≥ 1 and padj ≤ 0.05). Then, Gene Ontology (GO) and Kyoto Encyclopedia of Genes and Genomes (KEGG) pathway enrichment analyses were performed using clusterProfiler (3.8.1) software to gain insights into the functions and pathways of these differentially expressed genes in biological processes. Furthermore, targeted protein–protein interaction (PPI) analysis of the differentially expressed genes was also conducted utilizing the STRING database based on the KEGG analysis. Finally, the obtained interaction network data were visualized using Cytoscape 3.10, providing a more intuitive understanding of the interactions and regulatory relationships of these genes within the cell.

### 4.10. Statistical Analysis

Data analysis was performed using one-way ANOVA and *t*-tests in GraphPad Prism 10. Then, graphs were generated and labeled. Statistical significance was determined at *p* < 0.05 (*), *p* < 0.01 (**), *p* < 0.001 (***), and *p* < 0.0001 (****).

## 5. Conclusions

In this study, in vitro co-incubation of PBMCs revealed the effect of *Fg*BMP-1 in the inhibition of proliferation, migration, apoptosis, and phagocytosis, the enhancement of NO production, and altered cytokine transcriptions, specifically elevating IL-4 and TGF-β and decreasing IFN-γ and IL-10. The transcriptomic analysis unveiled the pivotal role of *Fg*BMP-1 in regulating cellular functions such as cell cycle and apoptosis, as well as its involvement in related biological processes, including host pathology and cancer. This study provides guidance for *Fg*BMP-1-regulatory mechanism exploration and contributes to elucidating the mechanisms involved in immune evasion of *Fasciola* species.

## Figures and Tables

**Figure 1:**
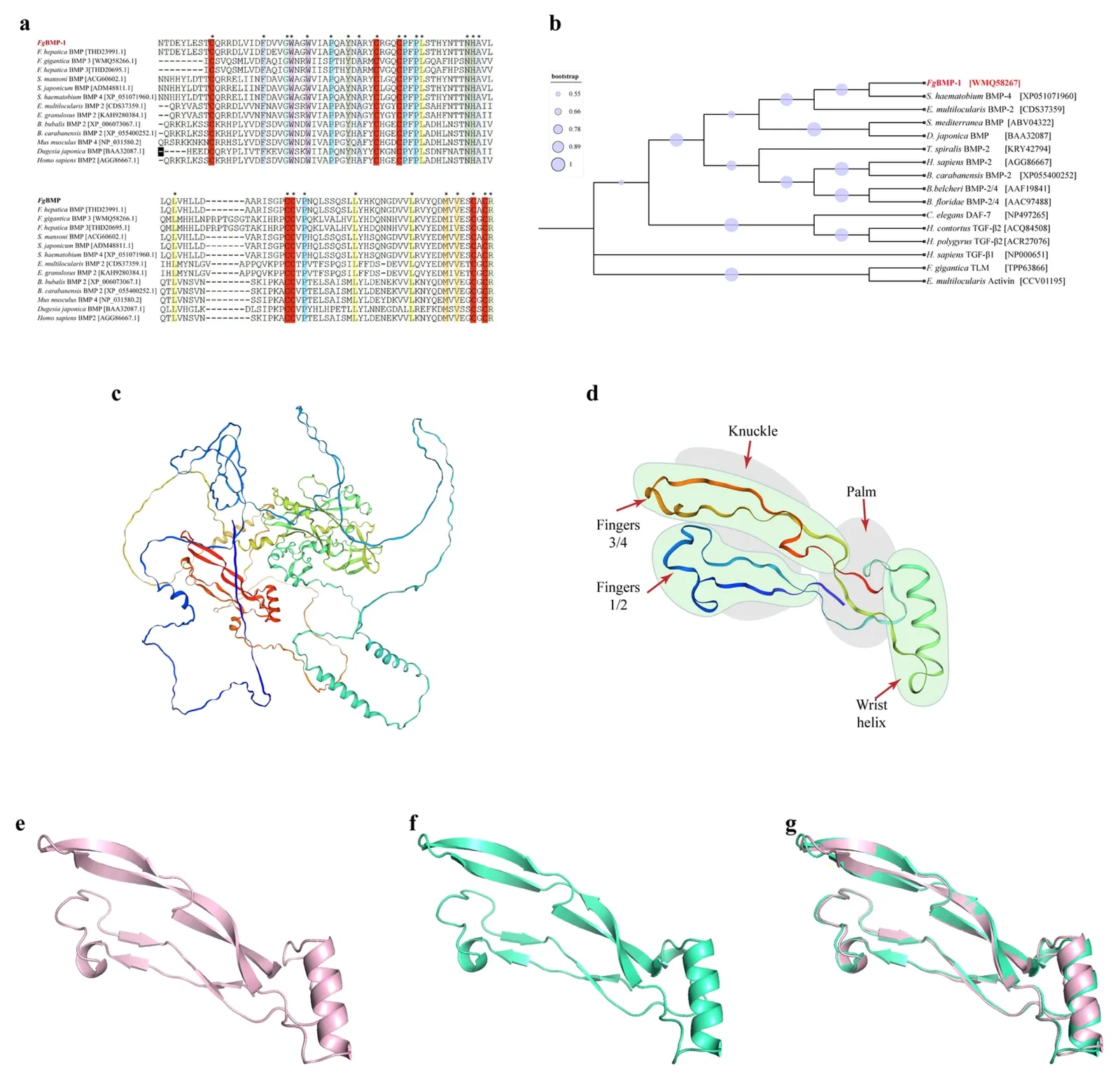
Sequence analysis and structural modeling of *Fg*BMP-1. (**a**) Amino acid multiple sequence comparison of the BMP ligands across species. Stars (*) indicate conserved residues. (**b**) Phylogenetic tree of *Fg*BMP-1. (**c**) Homology modeling of *Fg*BMP-1 precursor. (**d**) Homology modeling of *Fg*BMP-1 mature monomer. (**e**) Structural alignment of *Bb*BMP-2. (**f**) Structural alignment of *Fg*BMP-1. (**g**) Structural alignment of *Bb*BMP-2 and *Fg*BMP-1.

**Figure 2:**
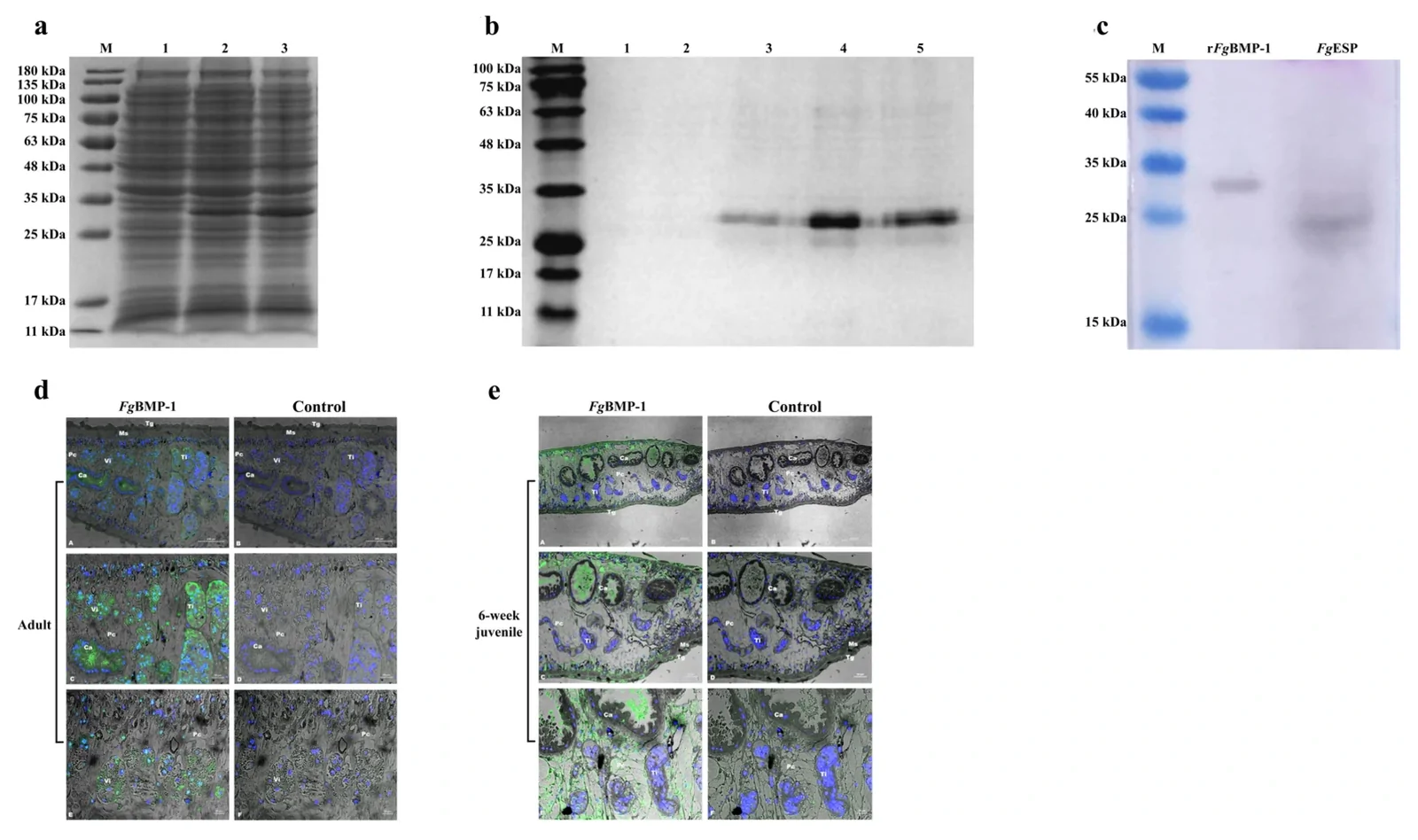
Protein expression and immunological localization of r*Fg*BMP-1. (**a**) SDS-PAGE analysis of r*Fg*BMP-1 expression. M: protein molecular weight standards; 1. empty plasmid control; 2. uninduced control; 3. induced cell lysate. (**b**) Purification of r*Fg*BMP-1. M: protein molecular weight standards; 1–5. eluent with 40 mM, 80 mM, 100 mM, 200 mM, 500 mM imidazole. (**c**) Western blot analysis of r*Fg*BMP-1 presence in *Fg*ESP. (**d**) Immunolocalization of *Fg*BMP-1 in *F. gigantica* adults. Ms: muscle; Tg: tegument; Pc: parenchyma; Ca: cecum; Vi: vitellaria; Ti: testis. scale bar: (A,B): 200 μm, (C,D): 50 μm, (E,F) 20 μm. (**e**) Immunolocalization of *Fg*BMP-1 in *F. gigantica* 6-week juveniles. scale bar: (A,B): 200 μm, (C,D): 50 μm, (E,F) 20 μm.

**Figure 3:**
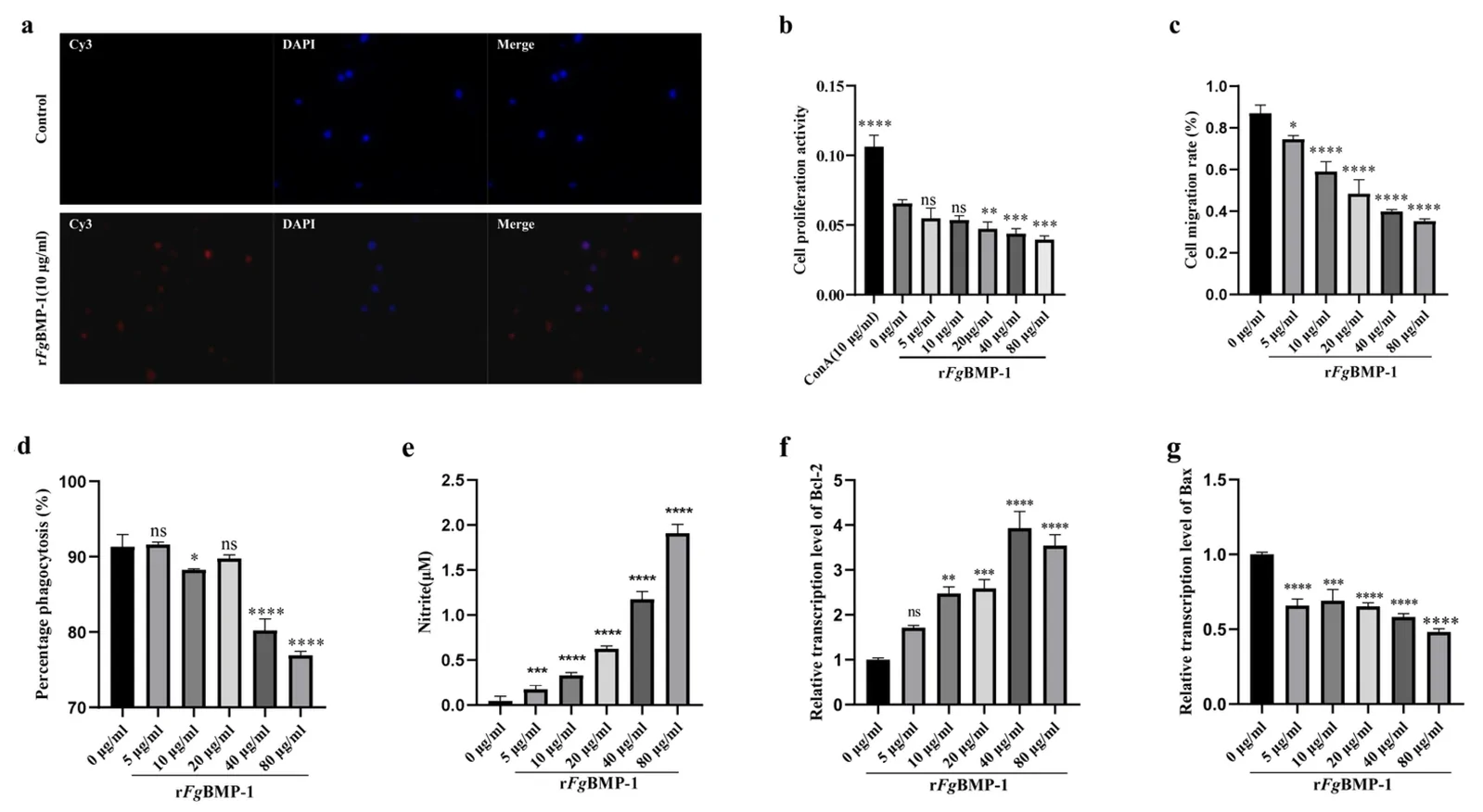
r*Fg*BMP-1 bind to the PBMCs and regulate its proliferation, migration and phagocytosis, promoted NO production. (**a**) Binding of r*Fg*BMP-1 to buffalo PBMCs detected by indirect immunofluorescence. (Top) Control: PBMCs incubated with anti-r*Fg*BMP-1 primary antibody and Cy3-conjugated secondary antibody (no r*Fg*BMP-1 stimulation). (Bottom) PBMCs stimulated with 10 µg/mL r*Fg*BMP-1, followed by incubation with mouse anti-r*Fg*BMP-1 monoclonal antibody and Cy3-conjugated goat anti-mouse IgG secondary antibody. (**b**) Effect of r*Fg*BMP-1 on the proliferation of buffalo PBMCs. (**c**) Effect of r*Fg*BMP-1 on the migration of buffalo PBMCs. (**d**) Effect of r*Fg*BMP-1 on the phagocytosis of buffalo PBMCs. (**e**). Effect of r*Fg*BMP-1 on the NO production of buffalo PBMCs. (**f**) Effect of r*Fg*BMP-1 on the transcription of apoptosis-related genes (*Bcl-2*) in buffalo PBMCs. (**g**) Effect of r*Fg*BMP-1 on the transcription of apoptosis-related genes (*Bax*) in buffalo PBMCs. * *p* < 0.05, ** *p* < 0.01, *** *p* < 0.001 and **** *p* < 0.0001.

**Figure 4:**
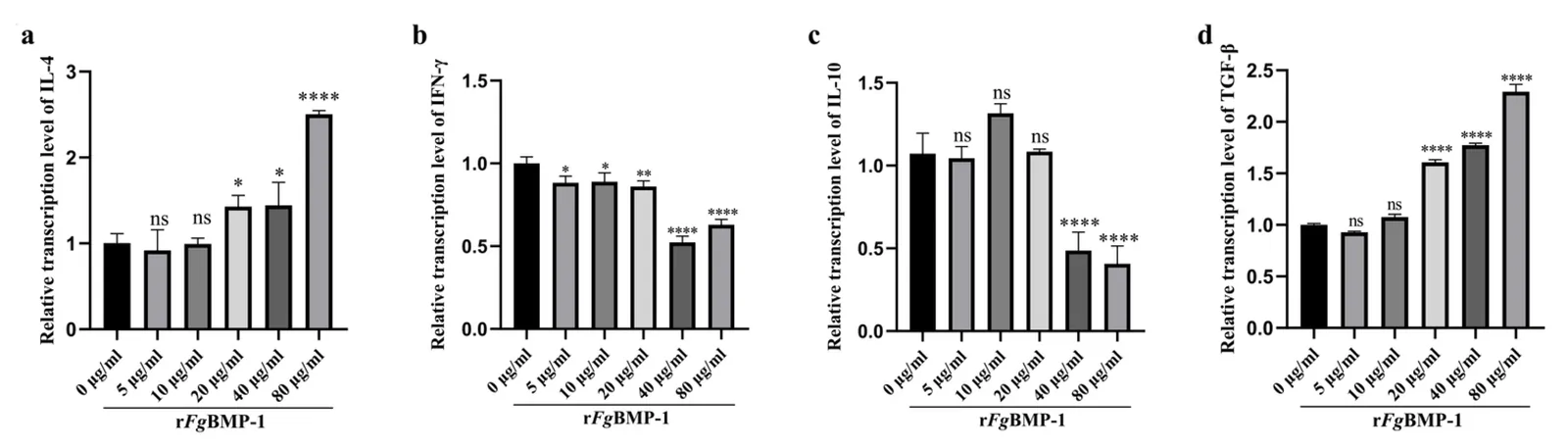
Effect of r*Fg*BMP-1 on the transcription of cytokines in buffalo PBMCs. (**a**) Effect of r*Fg*BMP-1 on IL-4 transcription. (**b**) Effect of r*Fg*BMP-1 on IFN-γ transcription. (**c**) Effect of r*Fg*BMP-1 on IL-10 transcription. (**d**) Effect of r*Fg*BMP-1 on TGF- β transcription. * *p* < 0.05, ** *p* < 0.01, and **** *p* < 0.0001.

**Figure 5:**
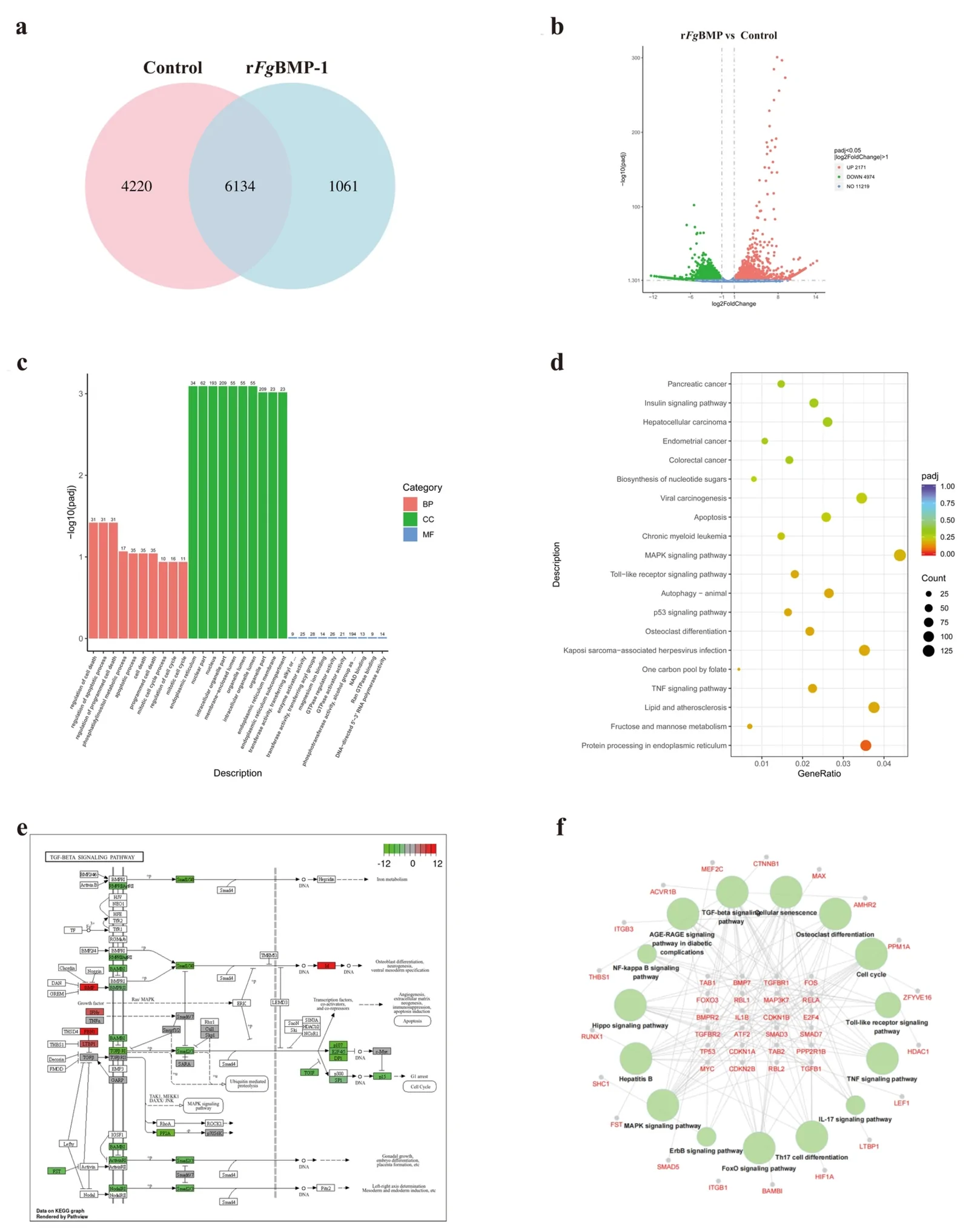
Transcriptomic analysis of r*Fg*BMP-1-treated PBMCs. (**a**) A Venn diagram of the control group and r*Fg*BMP-treated group. (**b**) Volcano plot of differentially expressed genes. (**c**) Histogram of GO analysis of differentially expressed genes. (**d**) Scatterplot of KEGG enrichment for differentially expressed genes. (**e**) Color annotation of TGF-β pathway. (**f**). Analysis of protein–protein interaction of components in the TGF-β pathway with other pathways.

**Table 1:** Molecular docking of *Fg*BMP-1 and buffalo-derived TGF-β receptors.

Classification of Receptors	Receptor Name	Protein ID	Binding Energy (Kcal/mol)
*Bb*BMP Type I Receptor	BMPR1A	XP_025140662	−13.3
	BMPR1B	XP_006071722	−12.6
*Bb*BMP Type I I Receptor	ACVR1	XP_025132115	−5.8
	BMPR2	XP_006080613	−14.6
	ACVR2A	XP_006078707	−12.6
*Bb*TGF-β Type I Receptor	TGFβR1	NP_001277855	−1.5
*Bb*TGF-β Type I I Receptor	TGFβR2	XP_006046923	−17.9

## Data Availability

All data generated or analyzed during this study are included in this published article and its Supplementary Information.

## Supplementary Materials

The following supporting information can be downloaded at https://www.mdpi.com/article/10.3390/ijms27198552/s1.
