# Supplementary material for: Fasciolagigantica Recombinant Bone Morphogenetic Protein (FgBMP) Modulates Various Functions of Buffalo Peripheral Blood Mononuclear Cells
Source: Int J Mol Sci. 2026 Sep 24;27(19):8552. doi: 10.3390/ijms27198552 (PMC13664918; doi:10.3390/ijms27198552)
Supplement: Supplementary file 1 [file ijms-27-08552-s001.zip › ijms-4553106-supplementary.pdf]

Table S1. Primer sequences used for PCR and RT-PCR analysis in this study.

Primer Name	Sequence(5'-3')
*Fg*BMP-1F	ATGTCTGGATTGTCTACTACTACT
*Fg*BMP-1R	CTACCGGCATGCGCAGCTTT
*Fg*BMP-2F	CCGAATTCGAGCTCCACTACGAAGAAACAAAACCAT
*Fg*BMP-2R	TGCGGCCGCAAGCTTACCGGCATGCGCAGCTTTCAACAA
pET28a-F	GGAAGCTGAGTTGGCTGC
pET28a-R	TCGATCCTCTACGCCGGACGC
GADPH-F	CCTGCACCACCAACTGCTTG
GADPH-R	TTGAGCTCAGGGATGACCTT
IL-4-F	CAGCATGGAGCTGCCT
IL-4-R	ACAGAACAGGTCTTGCTTGC
IL-10-F	CTGTGCCTCTCCCCTAGAGT
IL-10-R	GCAGCTAGCTCCACAAGGAA
IFN-ϒ-F	GTCTCCTTCTACTTCAAACT
IFN-ϒ-R	ATTCTGACTTCTCTTCCGCT
TGF-β-F	CGTGCTAATGGTGGAATAC
TGF-β-R	GCCAGGAATTGTTGCTATA
Bax-F	TTTCTGACGGCAACTTCAAC
Bax-R	GGGTGTCCCAAAGTAGGAGAG
Bcl-2-F	TCAATTGTCGTGGCATCAAAA
Bcl-2-R	CCCCCGACACCTGTTAGCTT
